# Analysis of 22 Posterior Ulnar Recurrent Artery Perforator Flaps: A Type of Proximal Ulnar Perforator Flap

**Published:** 2009-12-16

**Authors:** Musa A. Mateev, Leonid Trunov, Hiko Hyakusoku, Rei Ogawa

**Affiliations:** ^a^ Department of Plastic, Reconstructive Microsurgery and Hand Surgery, National Hospital of Kyrgyzstan, Bishkek, Kyrgyzstan; ^b^ Department of Plastic, Reconstructive and Aesthetic Surgery, Nippon Medical School, Tokyo, Japan

## Abstract

**Background:** The proximal ulnar artery has several branches, including perforators that are directly derived from the ulnar artery and anterior/posterior recurrent arteries. There are only a few reports of flaps that use the anterior/posterior recurrent arteries, and flaps employing their perforators as a main pedicle are yet to be reported. In this study, posterior ulnar recurrent artery perforator (PURAP) flaps were employed for elbow and forearm reconstruction. **Methods:** The 22 cases of reconstruction by PURAP flaps were analyzed in terms of the cause of injury, the recipient site, the vascular pedicle of the flap, flap size and survival, and the quality of the outcome. Donor-site morbidity, including the development of scars and numbness, was also evaluated. **Results:** All flaps were vascular pedicled island flaps. The perforator used was the medial and posterior perforator in 14 (63.6%) and 8 (36.4%) cases, respectively. The average flap size was 10 × 5 cm. Six months after the operation, the outcomes were judged to be excellent in 15 cases (68.2%), good in 6 cases (27.3%), and poor in 1 case (4.5%) because of partial necrosis of the distal part of the flap. **Conclusions:** PURAP flaps can be harvested with 2 types of perforator pedicles (the medial or posterior perforator) and offer greater safety and flexibility, and less donor-site morbidity, than existing flaps used for elbow and forearm reconstruction. The ability to close the donor site primarily is a significant benefit of this flap.

There are 4 to 9 perforators that travel from the ulnar artery, the majority of which (69%) are musclocutaneous perforators that penetrate the flexor carpi ulnaris muscle.^1^ Ulnar artery perforator flaps can be classified into 3 types according to where the perforators originate, namely, proximal ulnar perforator flaps, middle ulnar perforator flaps, and distal ulnar perforator flaps.^2^ Of these 3 types of perforator flaps, we have focused our study on proximal ulnar perforator flaps. Previous reports indicate that there are several branches coming from the proximal ulnar artery, including 2 perforators that are directly derived from the ulnar artery, namely, the anterior recurrent and posterior recurrent arteries (Fig 1a).

El-Khatib et al^3^ have reported a pedicled adipofascial flap based on the most proximal 2 to 4 perforators of the ulnar artery (located 1 to 5 cm from the origin of the artery). This is an example of flaps using perforators derived directly from the proximal ulnar artery. However, there are only a few reports of flaps that use the anterior/posterior recurrent artery,^4^–^7^ and flaps using these perforators as the main pedicle are yet to be reported. Here, we have analyzed the utility of posterior ulnar recurrent artery perforator (PURAP) flaps for elbow and forearm reconstruction.

## PATIENTS AND METHODS

### Analysis of patients

The elbow and forearm defects of 22 cases were reconstructed by PURAP flaps between 2000 and 2008. The patients were between 6 and 48 years of age. These cases were analyzed in terms of the cause of injury, the recipient site, the vascular pedicle of the flap, flap size, flap survival, and the quality of the outcome. The latter parameter was evaluated by 3 different plastic surgeons who designated the outcome as excellent, good, or poor on the basis of the color and texture matches between the flap and the recipient site. Moreover, donor site morbidity, including the development of scars and numbness, was also evaluated.

### Operative methods

**Figure 1:**
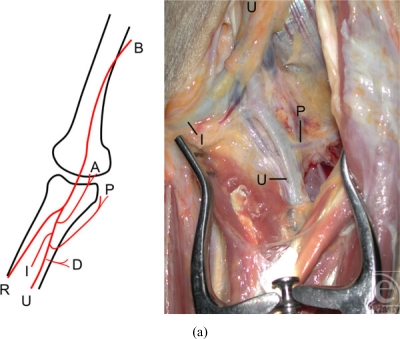

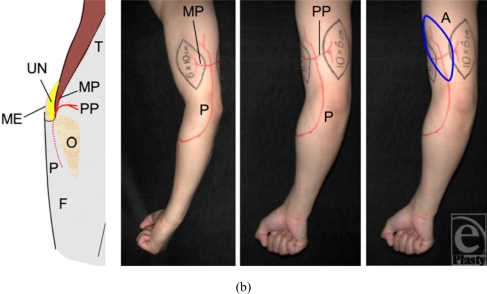
(a) A schema of the ulnar artery and its branches. The proximal large branches of the ulnar artery are the anterior and posterior ulnar recurrent arteries. The posterior ulnar recurrent artery is larger than the anterior ulnar recurrent artery and originates from the ulnar artery below it. It passes backward and medialward on the Flexor digitorum profundus, behind the Flexor digitorum sublimis, and ascends behind the medial epicondyle of the humerus. A: anterior ulnar recurrent artery; B: brachial artery; D: direct cutaneous perforator artery; I: interosseous artery; P: posterior ulnar recurrent artery; R: radial artery; and U: ulnar artery. (b) The PURAP flaps can be harvested with a perforator pedicle; thus, our 2 types of flaps (medial type and posterior type) are located more laterally than the conventional posterior ulnar recurrent artery flap (medial upper arm flap). Of the two PURAP flaps that are possible, we selected the more reliable flap intraoperativelly. The ability to close the donor site primarily is a significant benefit of this flap. Schemas of PURAP flaps and the conventional ulnar recurrent artery flap (medial upper arm flap). A: Conventional posterior ulnar recurrent artery flap (medial upper arm flap); F: flexor carpi ulnaris; ME: medial epicondyle; MP: medial perforator of posterior ulnar recurrent artery; O: olecranon; P: posterior ulnar recurrent artery; PP: posterior perforator of posterior ulnar recurrent artery; T: tricipital aponuerosis; and UN: ulnar nerve.

In all cases, the flaps were designed after debridement of wounds or complete resection of burn scars at the recipient sites. Generally, there are 2 perforators that originate from PURAP, namely, the medial and posterior perforators. Consequently, 2 flaps, one based on the medial perforator and one based on the posterior perforator, are possible. This flap is designed on the medial aspect of the posterior upper arm, immediately above the posterior ulnar recurrent artery in 2 locations depending on whether the medial or posterior perforator arising from the posterior ulnar recurrent artery is to be used. As a result, 2 flaps are designed before surgery, both of which are located more laterally than the conventional posterior ulnar recurrent artery flap (medial upper arm flap), which is located between the 2 PURAP flap types (Fig 1b). In all 22 cases, both flaps were designed on the medial and posterior sides of the distal upper arm, respectively, and the flap that seemed more reliable was then selected intraoperatively (Fig 1b).

Depending on the size and shape of the recipient site, a flap was designed such that the donor site could be closed primarily. In our experience, the elevation of a flap with a maximum width of 6 cm leaves a donor site that can be closed primarily without any problems. The length of the flaps ranged from 7 to 15 cm, with an average of 10–12 cm. In all cases, flap dissection was performed on a tourniquet. In 18 patients older than 21 years, only a brachial plexus block was used.

Flap elevation was initiated from the medial margin to identify the ulnar nerve, the posterior ulnar recurrent artery, and its medial and posterior perforators in the septocutaneous components. At this time, we determined which of the 2 perforators was larger and thus most suitable as the main pedicle of the flap. The lateral border of the selected flap was then incised, and the flap was islanded completely. All cutaneous veins were dissected and ligated. After elevation of the flap, the donor site was closed primarily while a tourniquet was applied.

## RESULTS

The lower arm defects of 22 patients were reconstructed by using the PURAP flap. All flaps were vascular pedicled island flaps. In 16 (72.7%) and 6 (27.3%) cases, the reconstruction was performed because of burns and traumatic wounds, respectively. The recipient sites were the cubital fossa in 16 cases (72.7%), the proximal forearm in 5 cases (22.7%), and the medial forearm in one case (4.5%). The perforators used for the PURAP flaps were medial in 14 cases (63.6%) and posterior in 8 cases (36.4%). The maximal size of the flap was 15 × 6 cm, with the average size being 10 × 5 cm. The maximal length of the vascular pedicle was 6 cm.

When the flaps were evaluated at least 6 months after the operation (maximum, 6 years), the outcomes were judged to be excellent in 15 cases (68.2%), good in 6 cases (27.3%), and poor in 1 case (4.5%) because of partial necrosis of the distal part of the flap. This distal necrosis epithelialized naturally without leaving outstanding scars. All patients whose outcomes were excellent or good were satisfied with the results (95.5%) and did not need any further operations.

## CASES

### Case 1 (Fig 2)

An 8-year-old boy suffered an electrical burn accident, and the soft tissues on the middle part of his forearm, including the skin, had necrotized completely. Six months later, the wound was reconstructed by a PURAP flap that was designed on his upper arm. Both medial and posterior perforators were detected preoperatively by Doppler ultrasonography and identified intraoperatively. The 2 perforators were similar in terms of vascular condition. The medial perforator was selected as the vascular pedicle because a flap based on this perforator would leave a more easily closed donor site. The length of the vascular pedicle was 6 cm and the flap size was 7 × 3 cm. The donor site could be closed primarily. The flap survived completely.

### Case 2 (Fig 3)

An injury to the right forearm of a 23-year-old man was inflicted by a rotation machine in a factory. The wound on the middle part of the forearm became deeply infected and necrosis developed on the proximal part of the forearm. While the original wound could be closed secondarily by direct suturing, the proximal wound could not be covered by the surrounding skin. Thus, the wound had to be reconstructed by a PURAP flap 2 months after the injury was sustained. The 2 perforators resembled each other intraoperatively in terms of vascular condition. The medial perforator was selected because a flap based on this perforator would leave a more easily closed donor site. The length of the medial perforator was 6 cm and the flap size was 14 × 6 cm. The donor site could be closed primarily. The flap survived completely.

### Case 3 (Fig 4)

A 21-year-old man suffered an amputation injury to the right forearm during a car crash. The distal end of the wound was reconstructed by a PURAP flap 3 weeks after the injury was sustained. The posterior perforator was larger and longer (6 cm) than the medial perforator (4 cm). Consequently, we used the posterior perforator. The size of flap was 7 × 4 cm, and the donor site was closed primarily. Complete flap survival was achieved.

## DISCUSSION

### Anatomy of the ulnar recurrent arteries and their perforators

The proximal large branches of the ulnar artery are the anterior and posterior ulnar recurrent arteries (Fig 1a). The anterior ulnar recurrent artery is also referred to as the epitrochlear artery.^8^ The anterior ulnar recurrent artery arises immediately below the elbow joint, runs upward between the Brachialis and Pronator teres, supplies twigs to those muscles, and, in front of the medial epicondyle, anastomoses with the superior and inferior ulnar collateral arteries.^9^ The posterior ulnar recurrent artery is much larger than the anterior ulnar recurrent artery and arises from the ulnar artery below it. It passes backward and medialward on the Flexor digitorum profundus, behind the Flexor digitorum sublimis, and ascends behind the medial epicondyle of the humerus. In the interval between this process and the olecranon, it lies beneath the Flexor carpi ulnaris, and ascending between the heads of that muscle (in relation to the ulnar nerve), it supplies the neighboring muscles and the elbow joint, and anastomoses with the superior and inferior ulnar collateral and the interosseous recurrent arteries.^9^ In our study, a perforator derived from the posterior ulnar recurrent artery was used for all 22 patients.

The recurrent arteries supply the medial aspect of the elbow (Fig 1b). Thomas et al^1^ reported that an average of 2 ± 1 perforators could be found in the ulnar recurrent artery territory. The mean vessel diameter was 0.8 ± 0.2 mm and the superficial length was 35 ± 12 mm.^1^ In our experience, we could harvest a maximum perforator pedicle length of 6 cm. All flaps in our 22 cases were vascular pedicled island flaps, but it may be possible to use these flaps as free flaps.

The average area^1^ supplied by each perforator is 63 ± 20 cm^2^ and the musculocutaneous/septocutaneous perforator ratio is 3:7.^1^ Prantl et al^7^ reported that the largest perforator of the posterior ulnar recurrent artery is generally located 10 cm proximal to the medial epicondyle. This description is compatible with our observations.

### Surgical indications for the PURAP flap

From a historical point of view, flaps based on the ulnar recurrent arteries include “recurrent flaps,”^10^ “medial upper arm fasciocutaneous flaps,”^5^ “ulnar recurrent fasciocutaneous^4^ or adipofascial^6^ flaps,” and “distal pedicled medial upper arm flaps.”^7^ Maruyama et al^4^ have reported elbow reconstruction, using an island fasciocutaneous flap based on the fasciocutaneous perforators of the ulnar recurrent vessels. Of the 10 cases that were reported, there was 1 case of sensory disturbance. This may reflect the proximity of the posterior ulnar recurrent artery to the ulnar nerve, and thus it is advisable that the ulnar nerve be identified before major dissection occurs. Maruyama et al have also reported adipofascial flaps that have been used to cover forearm wounds left after tumor resection.^6^ In addition, Bhattacharya et al^5^ have reported a free flap form of the distally based medial upper arm fasciocutaneous flap for use with palmar defects.

The flap described in this study is based on the posterior ulnar recurrent artery itself. During surgery, we select which of the 2 perforators seems more reliable (Fig 1b). We believe that these features of our PURAP flap mean it offers greater safety and flexibility and less donor-site morbidity. If much larger flaps are needed, it is possible to combine the 2 types of PURAP flap and harvest them using the posterior ulnar recurrent artery itself as the pedicle. In addition, this combined flap could be divided into 2 parts based on the medial and posterior perforators.

Indications for the use of the PURAP flap could also be upper limb defects. The proximal forearm, elbow, and the anterior aspect of the upper arm can be covered by a type of vascular pedicled island flap, while other regions on the upper limb could be reconstructed by a type of free flap. The PURAP flap is advantageous for upper limb reconstruction because of the color and texture match and the simplicity and speed of the operation (only a brachial plexus block is needed for ipsilateral reconstruction). That the donor site can be closed primarily is another significant benefit of this flap.

## Figures and Tables

**Figure 2:**
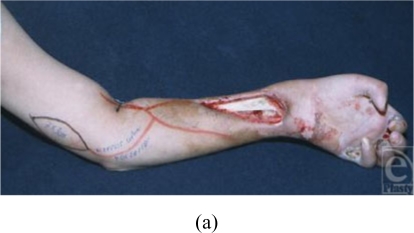

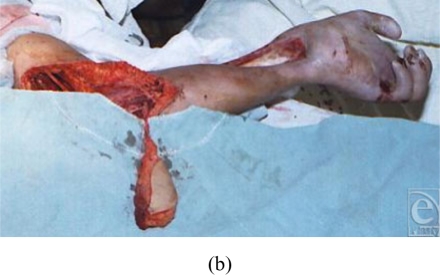

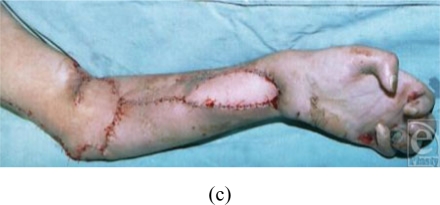

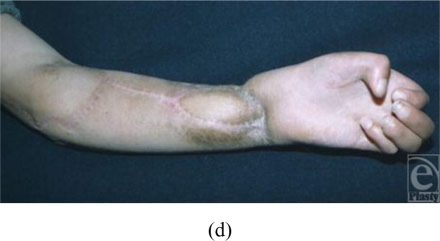
The electrical burn wound was reconstructed by the PURAP flap. The medial perforator was selected for this flap. The length of the vascular pedicle was 6 cm, and the flap size was 7 × 3 cm. The donor site could be closed primarily. The flap survived completely. (a) Preoperative view; (b) flap elevation; (c) view immediately after the operation; and (d) view 1 year after the operation.

**Figure 3:**
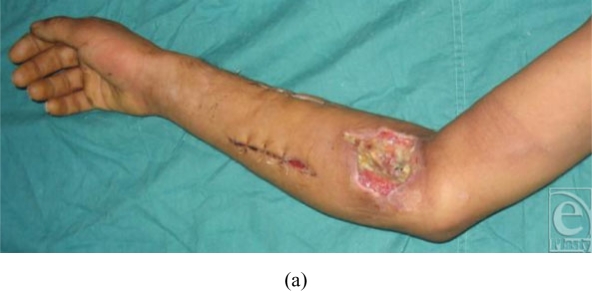

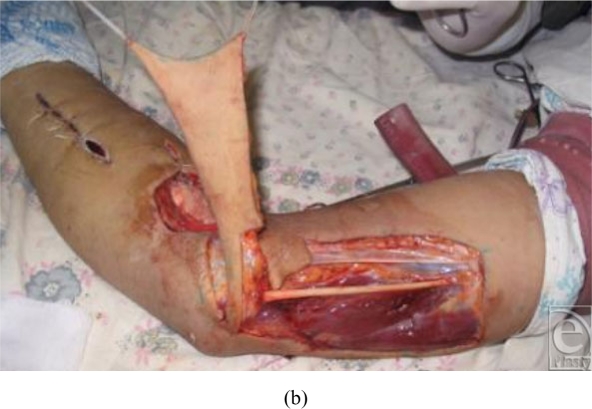

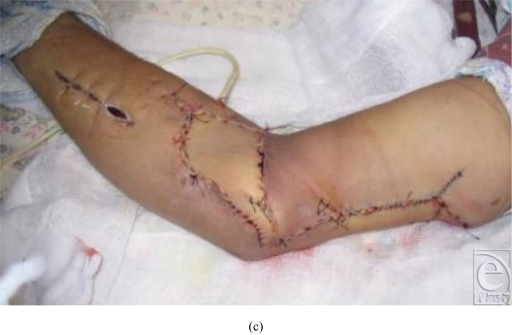

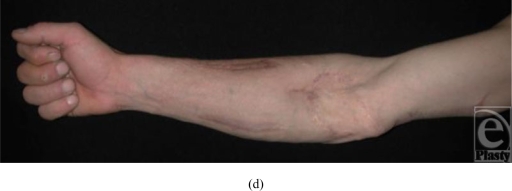

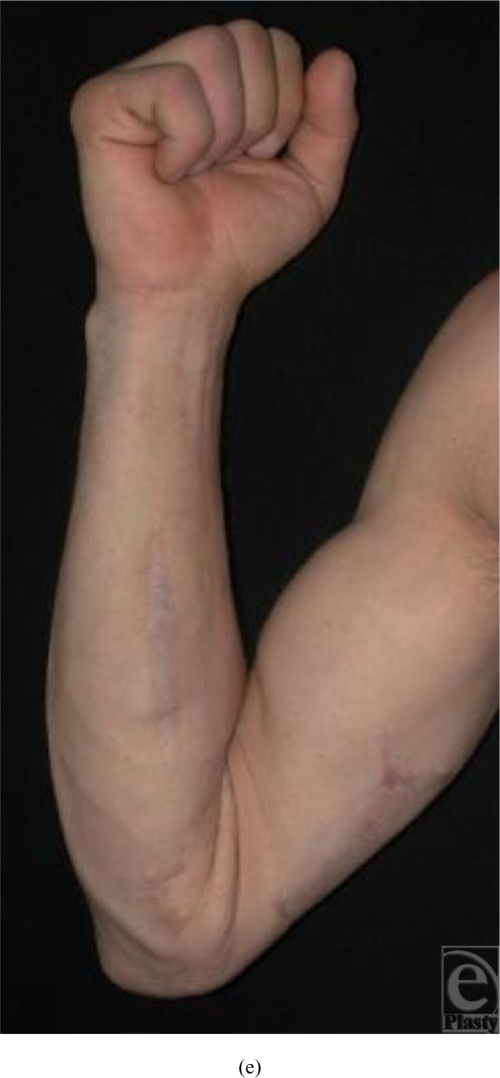
The right forearm became infected and had to be reconstructed by a PURAP flap. The medial perforator was selected for this flap. The length of the medial perforator was 6 cm. The flap size was 14 × 6 cm. The donor site could be closed primarily. The flap survived completely. (a) Preoperative view; (b) flap elevation and transfer; (c) view immediately after the operation; and (d, e) view 1 year after the operation.

**Figure 4:**
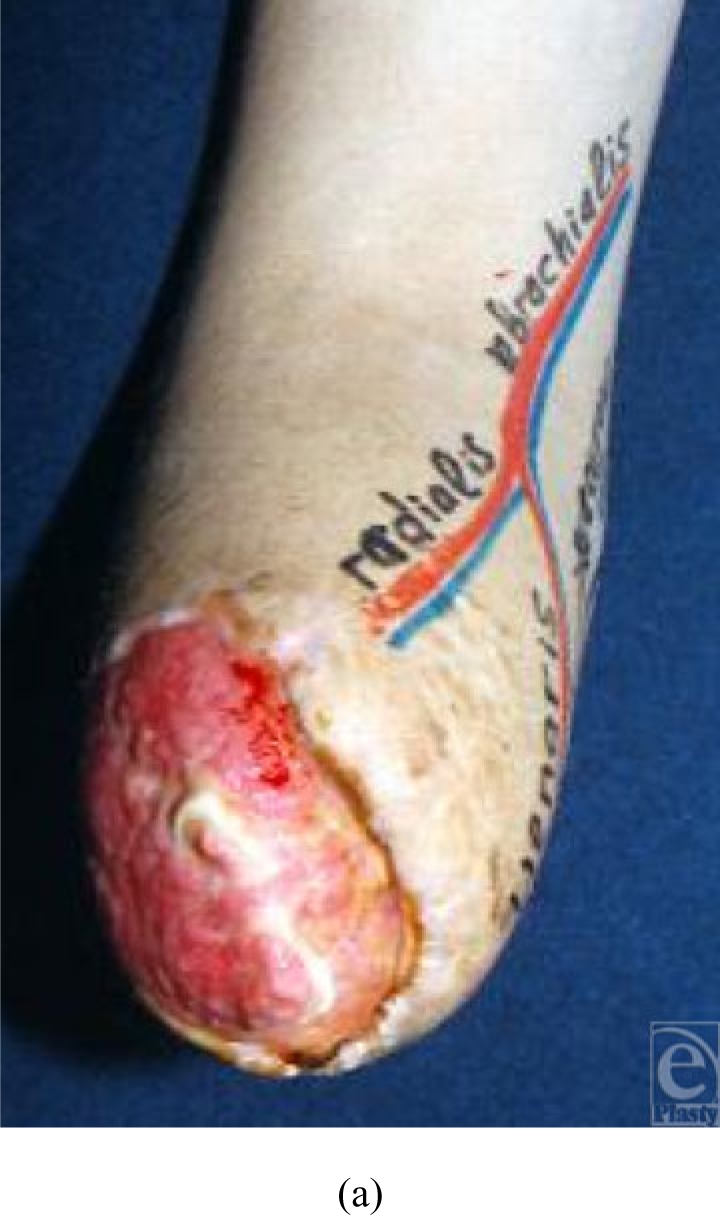

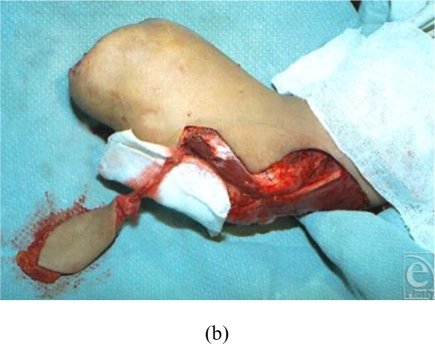

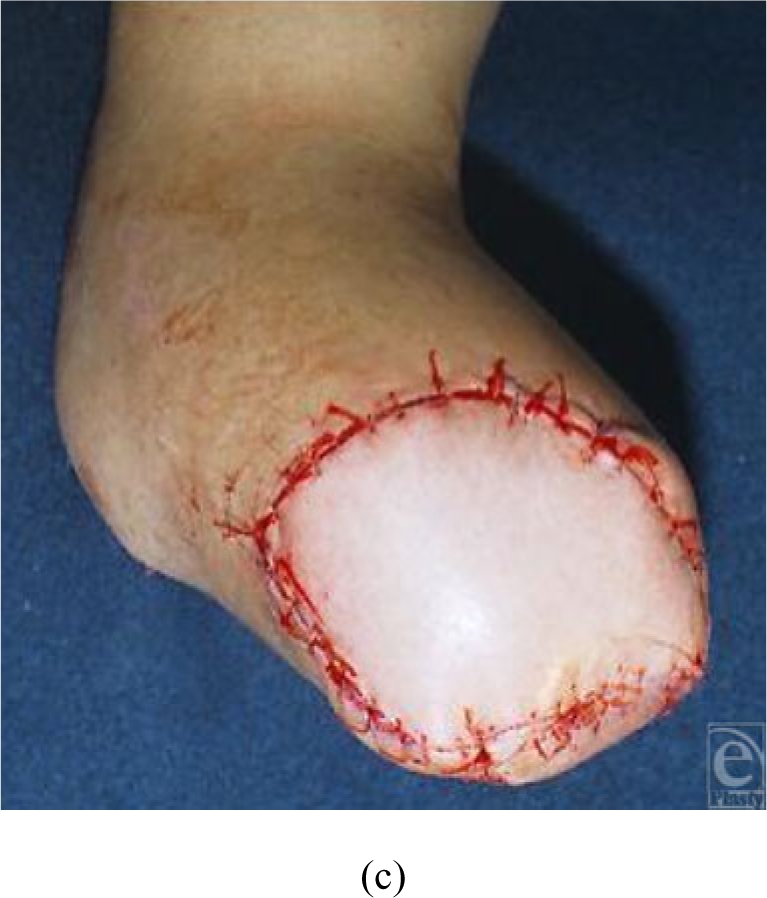

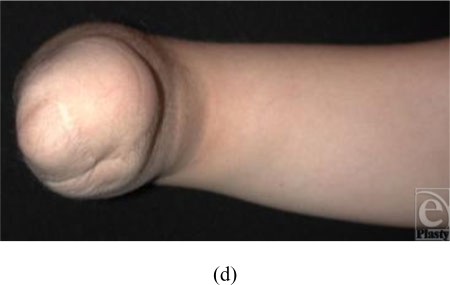

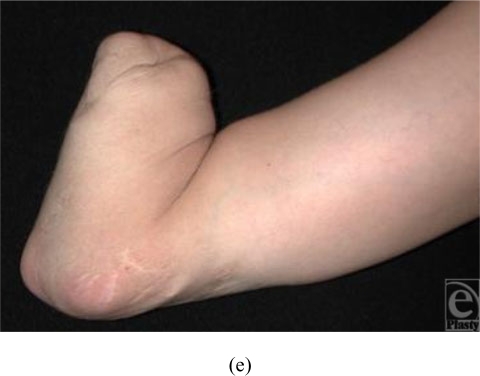

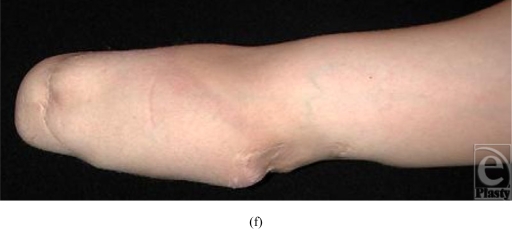

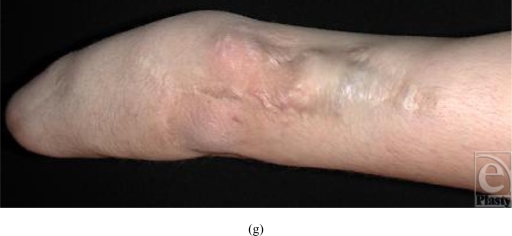
The right forearm suffered an amputation injury during a car crash and its distal end had to be reconstructed by a PURAP flap. The posterior perforator was larger and longer (6 cm) than the medial perforator (4 cm) and thus was used in this case. The size of the flap was 7 × 4 cm, and the donor site was closed primarily. Complete flap survival was achieved. (a) Preoperative view; (b) flap elevation and transfer; (c) view immediately after the operation; and (d–g) view 1 year after the operation.
